# Quantification of three-dimensional soft tissue artifacts in the canine hindlimb during passive stifle motion

**DOI:** 10.1186/s12917-018-1714-7

**Published:** 2018-12-07

**Authors:** Cheng-Chung Lin, Chia-Lin Chang, Ming Lu, Tung-Wu Lu, Ching-Ho Wu

**Affiliations:** 1 Department of Electrical Engineering, Fu Jen Catholic University, New Taipei City, Taiwan; 2 Institute of Veterinary Clinical Science, School of Veterinary Medicine, National Taiwan University, Taipei City, Taiwan; 3 Institute of Biomedical Engineering and Department of Orthopedic Surgery, School of Medicine, National Taiwan University, Taipei City, Taiwan

**Keywords:** 3-D marker displacements, CT, Fluoroscopy, Kinematics, Motion analysis, Soft tissue artifacts

## Abstract

**Background:**

Three-dimensional joint kinematics during canine locomotion are commonly measured using skin marker-based stereophotogrammetry technologies. However, marker-related errors caused by the displacement of the skin surface relative to the underlying bones (i.e., soft tissue artifacts, STA) may affect the accuracy of the measurements and obscure clinically relevant information. Few studies have assessed STA in canine limbs during kinematic analysis. The magnitudes and patterns of the STA and their influence on kinematic analysis remain unclear. Therefore, the current study aims to quantify the in vivo STA of skin markers on the canine thigh and crus during passive joint motion. The stifle joints of ten dogs were passively extended while the skin markers were measured using a motion capture system, and skeletal kinematics were determined using a CT-to-fluoroscopic image registration method.

**Results:**

The skin markers exhibited considerable STA relative to the underlying bones, with a peak amplitude of 27.4 mm for thigh markers and 28.7 mm for crus markers; however, the amplitudes and displacement directions at different attachment sites were inconsistent. The markers on the cranial thigh and lateral crus closer to the stifle joint had greater STA amplitudes in comparison to those of other markers. Most markers had STA with linear and quadratic patterns against the stifle flexion angles. These STA resulted in underestimated flexion angles but overestimated adduction and internal rotation when the stifle was flexed to greater than 90°.

**Conclusions:**

Marker displacements relative to the underlying bones were prominent in the cranial aspect of the thigh and the proximal-lateral aspect of the crus. The calculated stifle kinematic variables were also affected by the STA. These findings can provide a reference for marker selection in canine motion analysis for similar motion tasks and clarify the relationship between STA patterns and stifle kinematics; the results may therefore contribute to the development of STA models and compensation techniques for canine motion analysis.

**Electronic supplementary material:**

The online version of this article (10.1186/s12917-018-1714-7) contains supplementary material, which is available to authorized users.

## Background

Joint biomechanics during canine locomotion has been measured mainly using kinematic or kinetic measurement units [1, 2]. The findings from these analyses are essential to understand the coordination of normal [3] and abnormal canine limb movements, which are associated with orthopedic abnormalities [4], and to evaluate treatment outcomes [5, 6]. Several quantitative tools have been developed for motion analysis. Optoelectrical stereophotogrammetry using skin markers [7] is commonly used in canine gait analysis [8]. However, marker-related errors that obscure clinically relevant information have been documented in human motion analysis [9]. These errors are caused by soft tissue artifacts (STA) that are characterized by the displacement of the skin surface relative to the underlying bone.

The amplitudes and patterns of STA displacements have been widely studied for human lower limbs [10], as have their cumulative effects on the calculated mechanical variables [11–13]. In this context, STA amplitudes are inconsistent among different marker locations [14], motion tasks [10], and subjects’ body characteristics [11] and are affected by several factors associated with stable and variable components [15]. The former factors are the result of soft tissue stretching and sliding in relation to the adjacent joint motion [16, 17], while the latter factors can be affected by the segmental deformation created during muscle activity [15] and possibly by the wobbling effects of soft tissues during vigorous activity. Joint rotations are the primary cause of STA in kinematics-driven STA models [16]; this implies that a knowledge of the relationships between STA and adjacent joint angles will facilitate the development of artifact compensation techniques and improve the estimation of segmental kinematics using skin markers.

To our knowledge, few studies have assessed STA in canine limbs during kinematic analysis. For example, skin movement relative to the underlying bones was revealed by changes in segment lengths calculated from skin marker positions during gait [18, 19]. In addition, two-dimensional (2-D) displacements of radiopaque markers were detected relative to the underlying anatomical landmarks using fluoroscopic images [18]. However, three-dimensional (3-D) marker displacements relative to the underlying bones must be determined using 3-D “STA-free” bone poses. Thus, a model-based tracking method using fluoroscopy and computerized bone models [20] appears to be a feasible solution for measuring canine skeletal kinematics [21].

The present study aimed to quantify the in vivo amplitudes and patterns of 3-D STA for markers on the craniolateral aspect of dogs’ thigh and crus during passive stifle extension. This goal was achieved using an integrated measurement unit that consisted of a C-arm for fluoroscopy and an infrared motion capture system. The cumulative effects of the STA on the calculated passive stifle kinematics were also evaluated.

## Methods

### Animals

This study included 10 client-owned, adult, mixed-breed dogs (age: 47.9 ± 26.4 months; weight: 21.76 ± 2.9 kg; body condition score: 5–6/9) without any detectable abnormalities on hindlimb radiographs and physical examinations, which were performed by two veterinary surgeons (CLC and CHW). The owners provided written informed consent for the data collection, and the study protocol was approved by Institutional Animal Care and Use Committee of National Taiwan University (Approval No: NTU104-EL-00086).

### Skin markers

**Fig. 1:**
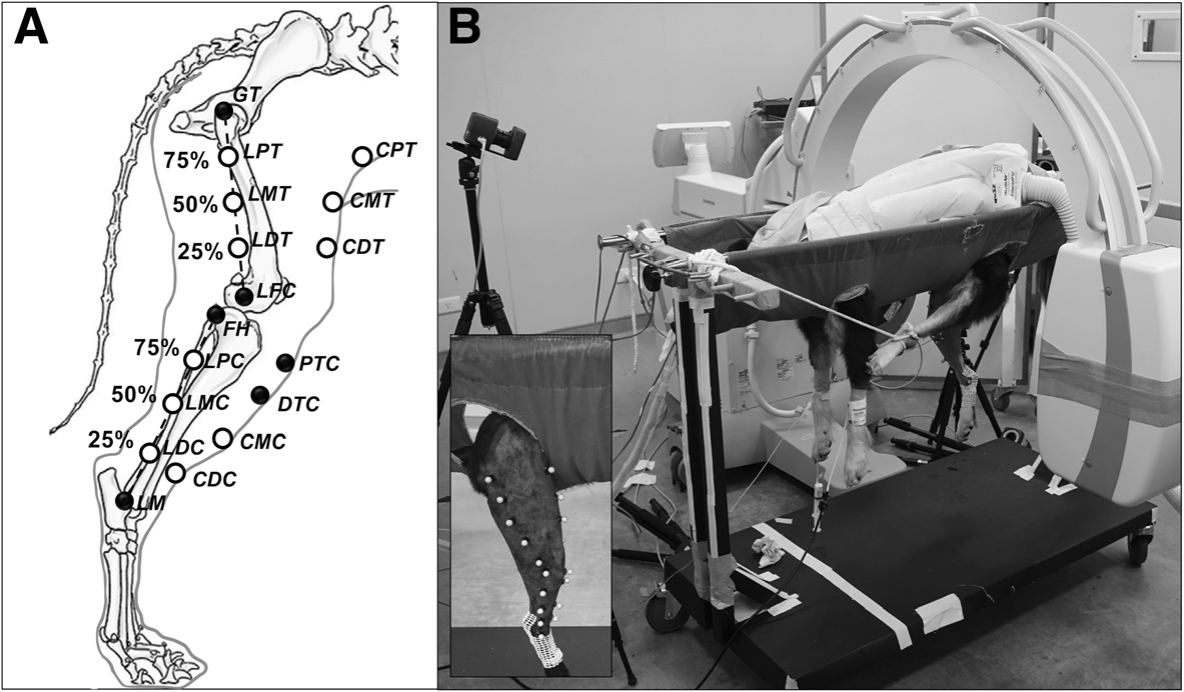
Marker attachment sites and experimental environment. (**a**) The attachment sites for skin markers on the right thigh and crus. The black solid circles indicate bony landmarks, and the hollow circles indicate technical markers. The lateral technical markers were evenly distributed between the two bony landmarks at an interval of 25% of the total distance. (**b**) The placement of the dog in a purpose-built hammock with a C-arm for fluoroscopy and infrared cameras for the motion capture system. The hammock was built to sustain the dog’s posture, and four openings were created to allow the limbs to hang down with minimal resistance

A total of 19 spherical, infrared-reflective markers (diameter: 8 mm) were placed on the skin surface of the canine right hindlimbs, including 9 markers placed on the thigh and 10 on the crus. Eight markers were placed on bony landmarks: the greater trochanter (GT), lateral femoral epicondyle (LFC), medial femoral epicondyle (MFC), fibular head (FH), proximal tibial crest (PTC), distal tibial crest (DTC), lateral malleolus (LM), and medial malleolus (MM). These landmarks were chosen to create a coordinate system for the thigh and crus [22]. Technical markers were placed on the cranial and lateral aspects of the thigh and crus, respectively (Fig. 1A). Three lateral thigh markers (LPT, LMT, and LDT) were evenly distributed along the axis connecting the GT and LFC at an interval of 25% of the distance between the GT and LFC. Three cranial thigh markers (CPT, CMT, and CDT) were placed over the cranial skin surface with level heights that were identical to those of the corresponding lateral markers. The crus technical markers were placed in the same manner, except for the cranial and proximal markers, as the tibial crest markers occupied that region.

Before marker placement, local hair was clipped to reduce its effect on the experimental data. All markers were subsequently attached by one of the authors (CLC) using cyanoacrylate and an over-layer of adhesive tape while the dogs stood still on the ground. In the standing posture, the angles of the stifle and hock joints were measured by CLC using goniometers. The locations of the marker points were selected to ensure that the markers were visible to the surrounding cameras during the test activities and based on the principles of marker point placement [23].

### Data acquisition

The C-arm fluoroscopy system (Arcadis Avantic; Siemens AG, Germany) was calibrated for the intrinsic parameters of the projection model and the fluoroscopic coordinate system (*F*_*cs*_); these parameters were obtained using a radiolucent calibration object comprising two parallel plates with lead markers embedded at defined positions. Six infrared cameras (Bonita 10; VICON, UK) were placed surrounding the isocenter of the C-arm. Six non-coplanar infrared markers attached to the calibration object at predefined positions were used to determine the transformation ${\mathrm{T}}_{L_{cs}}^{F_{cs}}$, which spatially registered the laboratory coordinate system (*L*_*cs*_) provided by the motion capture system to the *F*_*cs*_ using a “singular variable decomposition” method [24]. Based on the transformation, the ensemble average of residual errors over the markers was < 0.76 mm.

General anesthesia was induced in the dogs using propofol (4–6 mg/kg; Lipuro 1%; B. Braun Melsungen AG, Germany). After intubation, anesthesia was maintained by isoflurane (Attane; Panion & BF Biotech Inc., Taiwan, ROC). Before the data collection, the dogs were positioned in a purpose-built hammock created using an aluminum trolley (Fig. 1B) such that the right stifle joint was located approximately in the center of the fluoroscopy field of view (FOV) (Fig. 1B), and the left leg was tied forward to allow the right leg to be imaged in isolation. The hock joint was kept at a fixed angle from the standing position using a custom-made plastic jig (Vet-Lite; Veterinary Specialty Products, Florida, USA). To minimize the resistance and skin stretching by the hammock, four openings in the hammock were made as large as possible to enable the limbs to hang down naturally while maintaining the necessary support and stability for the dogs. CLC and CHW both verified that no skin was accidentally stretched before the data collection began.

The tested stifle joint was carefully flexed to the extreme flexion position (the starting joint angle) by a veterinary surgeon and held by a cotton rope linking the hammock and canine hindfoot. The rope was then released at a controlled speed from the hammock edge, which returned the stifle to extension with the limb naturally hanging down (the ending joint angle) over a 4 s interval. During stifle extension, the fluoroscopy system (frame rate: 30 fps, tube voltage: 50–53 kVp, tube current: 0.3–0.6 mA) and the motion capture system (sampling rate: 120 fps) simultaneously collected mediolateral fluoroscopic images (FOV: 300 mm in diameter; resolution: 1024 × 1024 pixels) and skin marker coordinates, respectively. Data were collected for three successful trials of passive extension for each dog. A static calibration trial was also performed in which the stifle joint was manually flexed to the subject-specific stifle angles from the standing position. A motion capture software (Nexus, VICON, UK) was used to reconstruct, process and label the marker data which were then extracted once for every four data points in the timeline for subsequent STA analysis. In a previous fluoroscopic study at a higher tube voltage and current [21], the delivered ionizing radiation dose was estimated to be 0.036 μSv per image, yielding a total dose of < 0.02 mSv to the dogs in the present study (0.036 μSv × 120 frames × 4 trials).

Each dog also underwent a computed tomography (CT) scan (Activiom 16; Toshiba, Japan) of the right hindlimb from the cranial border of the ilium to the distal end of the foot (voxel size: 0.625 mm × 0.625 mm × 0.3 mm). During the CT scan, the dogs were positioned in ventral recumbency with the hindlimbs caudally extended. After data collection, all dogs were uneventfully recovered from anesthesia and they were discharged under care of their owners.

### Reconstruction of skeletal kinematics

The subject-specific CT data were semi-automatically segmented using a region-growing method for the femur and tibia/fibula. Voxels not involved in the segment region were given an intensity value of − 1000 (i.e., the Hounsfield unit value of air). A minimum bounding volume containing a complete bone segment was subsequently extracted from the original CT data set, which provided the voxel-based bone model. All image processing was performed using our self-developed software, which was implemented in MATLAB (MathWorks Inc., Natick, USA).

**Fig. 2:**
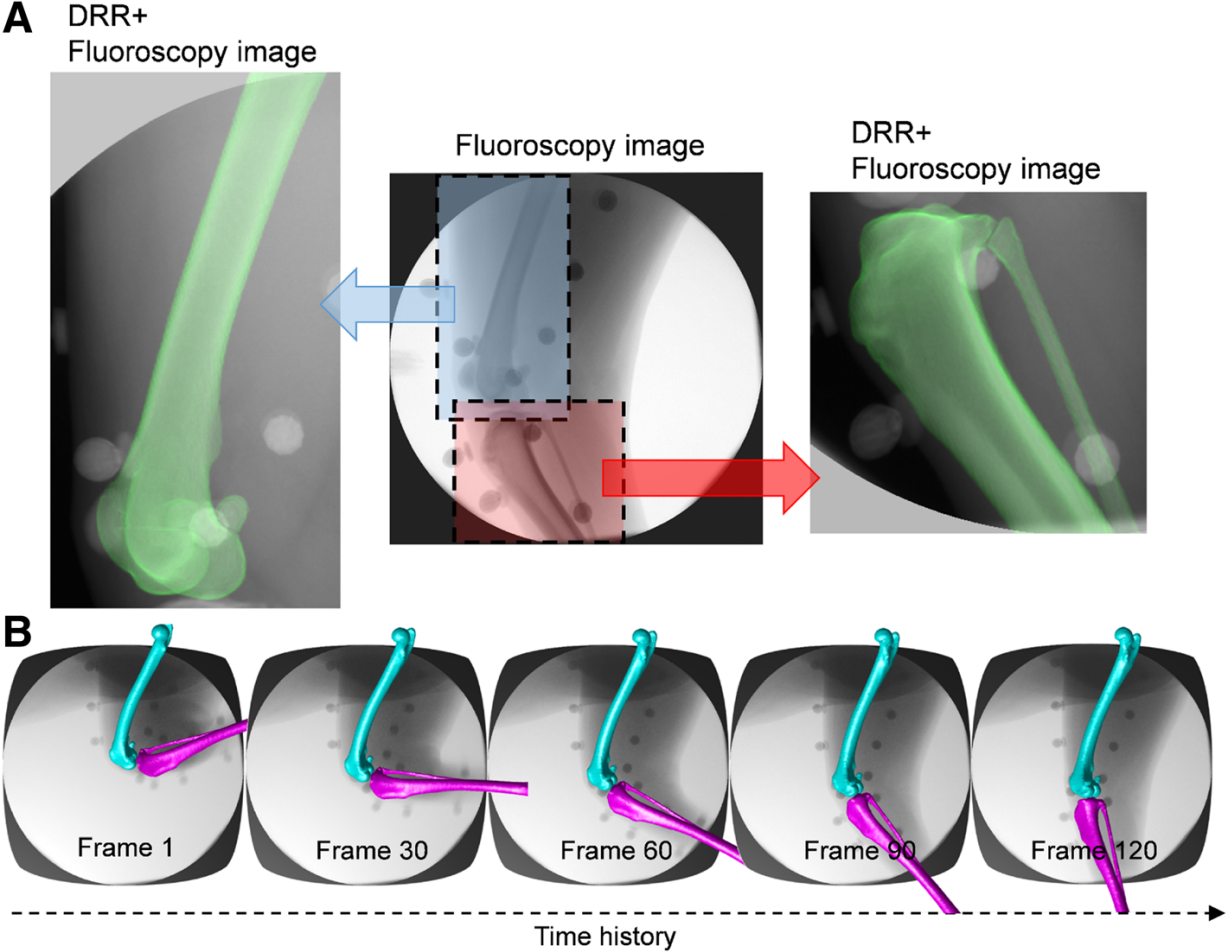
Model-based tracking method using fluoroscopy. (**a**) The three-dimensional poses of the femur and tibia/fibula were determined by best matching the digitally reconstructed radiography (DRR, green) and the corresponding fluoroscopic images (gray). (**b**) The same procedure can be applied to a full sequence of fluoroscopic images to reconstruct the three-dimensional kinematics of the stifle joint

Model-based tracking was used to determine the 3-D positions and orientations of the bone models, based on digitally reconstructed radiographs that were matched to the fluoroscopic images using a numerical optimization procedure (Fig. 2A) [20, 25]. The digitally reconstructed radiographs were created by projecting the voxel-based bone model onto the image plane using ray tracing and a tri-linear interpolation method [26]. The image matching was performed frame-by-frame throughout the entire fluoroscopy sequence, providing the kinematics of the bone model (Fig. 2B). The model-based tracking was performed using our self-developed software [26], which was implemented on a personal computer (Intel Core i7 860: 2.8 GHz, NVIDIA GeForce GTX TITAN X, and 16 GB of RAM).

### Soft tissue artifact analysis

#### STA at the marker level

**Fig. 3:**
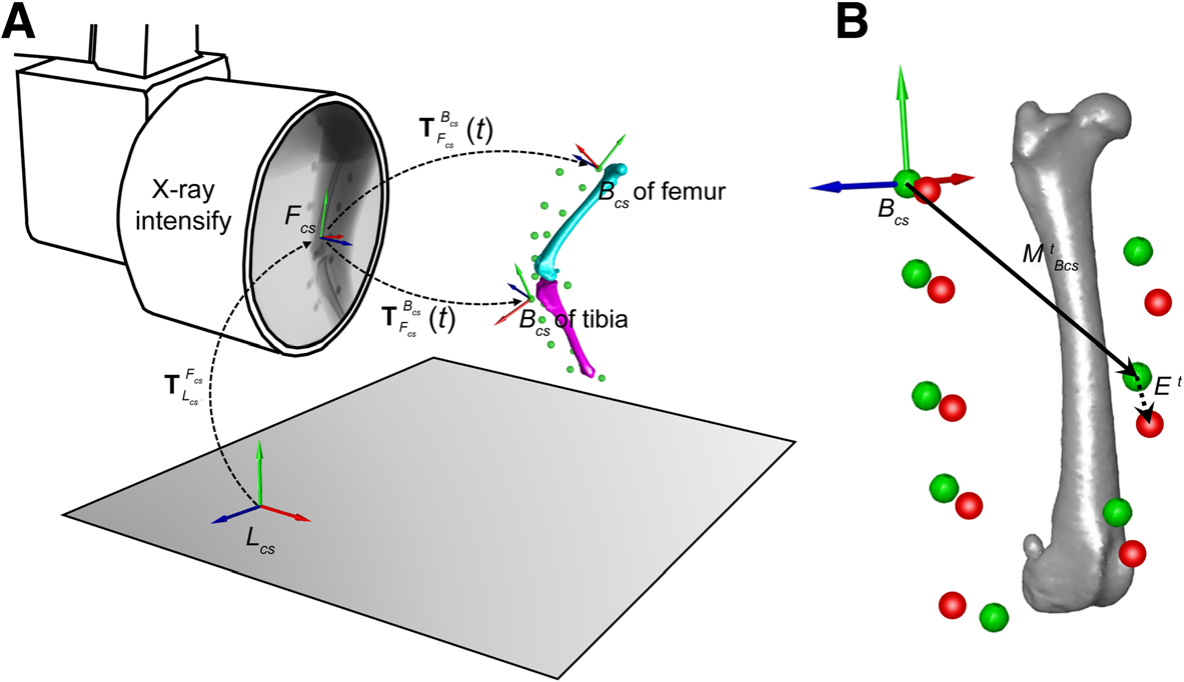
Analysis of the soft tissue artifacts. (**a**) A schematic representation of the coordinate transformation between the laboratory coordinate system (*L*_*cs*_), the fluoroscopy coordinate system (*F*_*cs*_), and the bone coordinate system (*B*_*cs*_). (**b**) Based on the marker position in *B*_*cs*_ during the calibration trial (green) and the position during the motion task (red), the marker displacement at frame *t* (i.e., *E*^*t*^: the soft tissue artifact at the marker level) is obtained by calculating the coordinate difference

The raw kinematics data were filtered using a fourth-order, low-pass Butterworth filter with a cutoff frequency of 6 Hz. Based on data from static calibration trial of each dog, a bone anatomical coordinate system, *B*_*cs*_, was created for the thigh and crus based on the registered pose of the femur and tibia and the coordinates of the bony landmark markers according to an established protocol (Fig. 3A) [22]. With the registered 3-D pose of the *B*_*cs*_ obtained from the model-based tracking (${T}_{F_{cs}}^{B_{cs}}$, Fig. 3A), the position vector of each marker in the corresponding *B*_*cs*_ at frame *t* was computed and denoted as ${M}_{B_{cs}}^t$ (Fig. 3B).

Based on the existence of an STA, ${M}_{B_{cs}}^t$ changed over time during the stifle extension task, and these displacements and variations over time were used to characterize the STA in the marker levels. Thus, the error vectors at frame *t* (*E*^*t*^, Fig. 3B) were determined as the difference in ${M}_{B_{cs}}^t$ from the motion task and the calibration trial, and this difference indicated the marker displacements relative to the underlying *B*_*cs*_ (Fig. 3B). The variation of ${M}_{B_{cs}}^t$ was estimated in terms of the total magnitude of the root-mean-square amplitude (*rmsd*) and the *rmsd* along each anatomical axis (*rmsd*_*c*_, where *c* represents the *x-*, *y-,* and *z-*axes) [27]. The maximal ranges of the marker displacement were quantified using the total magnitude of the peak-to-peak amplitude (Δ*p*_*max*_) and the peak-to-peak amplitude along each anatomical axis (Δ*p*_*c*_) [27]. The variables were initially averaged over three trials for each dog, and the mean and standard deviation (SD) for all dogs were calculated. All attached markers, except for the markers at MFC and MM, were included in the STA analysis. The two medial-side markers were used only for determining the anatomical coordinate system in the static calibration trial and were not used in the STA analysis during the passive motion trials, because the markers were normally invisible to the cameras at high stifle flexion angles.

The STA patterns relative to the stifle joint angles were evaluated by expressing each of the components of the error vectors of each marker relative to the stifle joint angles, which were then fitted with polynomial functions of different degrees. The polynomial regression functions with the highest adjusted R^2^ values were subsequently identified and accepted as the best-fitting curves for the error vectors. The linearity of the STA pattern against the stifle angles (linear, quadratic, or higher) was defined as the lowest-order polynomial that accounted for 90% of the R^2^ value of the best-fitted polynomial or as the lowest-order polynomial that had a standard regression error of < 1 mm.

#### STA at the joint level

The STA-free trajectories of the markers (i.e., “virtual markers”) were derived using the kinematics of the bones [11]. A cluster template for each segment, consisting of the local coordinates of the marker relative to the associated *B*_*cs*_, was determined during the static calibration trials. Based on 3-D coordinates of the skin or virtual markers and the cluster template, the segment positions and orientations were obtained using the pose estimator [24]. The stifle joint angles were then calculated using the relevant segment poses following a z–x–y Cardanic rotation sequence, which corresponded to flexion/extension (Flex/Ext), adduction/abduction (Add/Abd), and internal/external rotation (IR/ER) [28]. To report the joint angles, we employed a convention for 3-D motion analysis [21] in which the reported flexion/extension angle is 0° for full extension, with increasing values corresponding to stifle flexion.

The effects of the STA on the stifle joint kinematics were quantified throughout the entire motion task using the differences between the skin marker-determined (SM-determined) and virtual maker-determined (VM-determined) kinematics. A paired *t*-test with a significance level of 0.05 was used to compare the SM- and VM-determined Flex/Ext, Add/Abd, and IR/ER values. The statistical analyses were performed using MATLAB (MathWorks Inc., Natick, MA, USA).

## Results

**Fig. 4:**
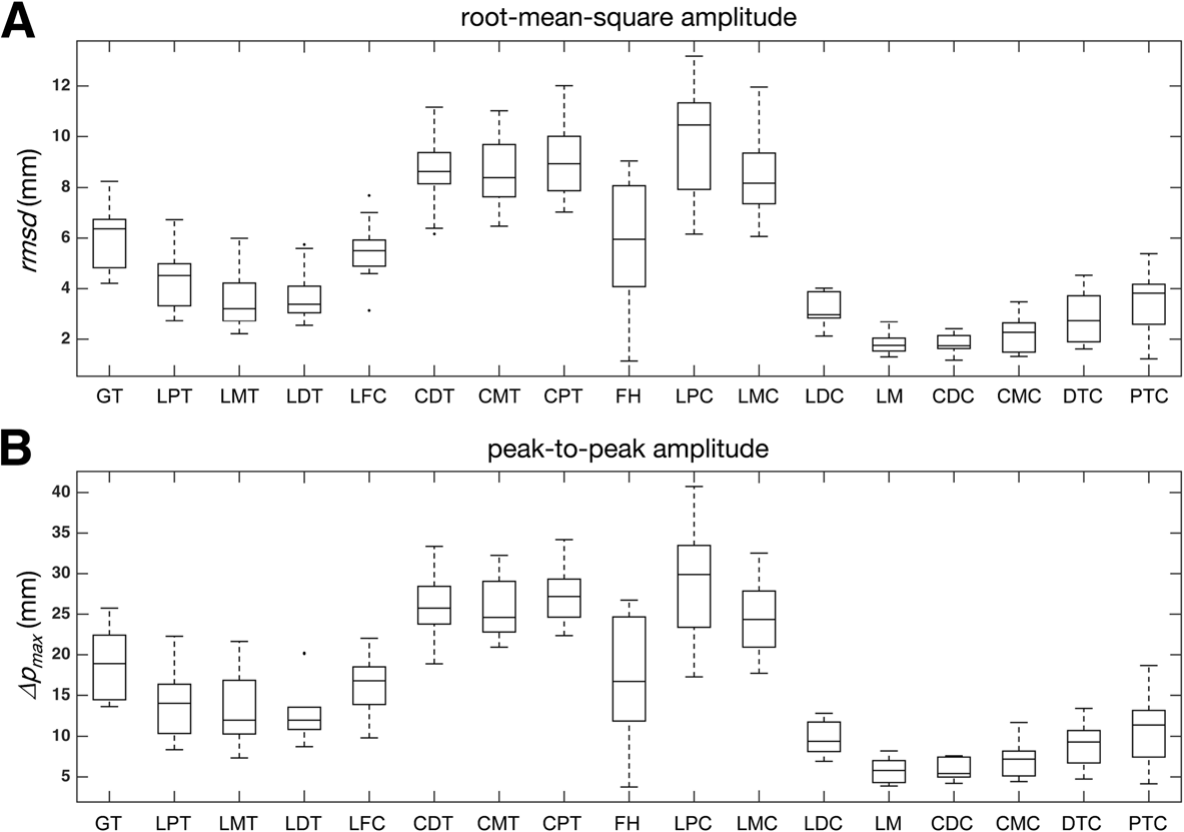
Box plot of the soft tissue artifacts of individual markers. The box plot of (**a**) the root-mean-square amplitude (*rmsd*) and (**b**) the peak-to-peak amplitude (∆*p*_*max*_) of the marker displacements relative to the underlying bone. The central lines indicate the median values, the top and bottom edges indicate the 75th and 25th percentiles of the error distributions, and the whiskers indicate the upper and lower extremes. Outliers are plotted as dots

**Fig. 5:**
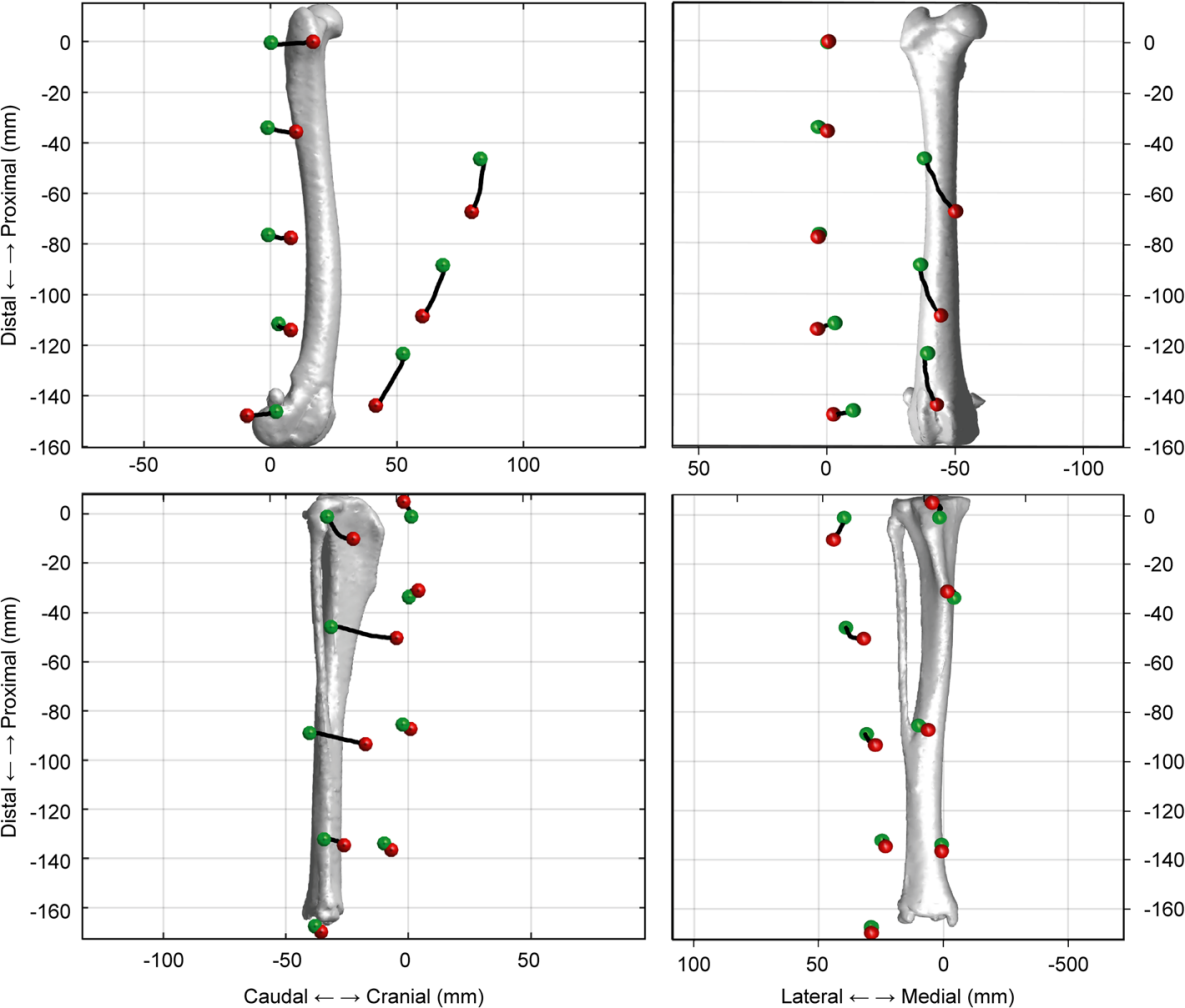
Three-dimensional displacements of the markers with respect to the underlying bones. The displacements of the skin markers (black line) when the stifle joint is extended (green) and at the fully flexed position (red). The data were averaged across all subjects

The means ± standard deviations of the stifle angle for the starting and ending joint angles were 147.6° ± 8.5° and 55.7° ± 12.5°, respectively. There were noticeable marker displacements relative to the underlying bones, with a maximum peak error amplitude (i.e., Δ*p*_*max*_) of 27.4 mm for thigh markers and 28.7 mm for crus markers (Table 1). Furthermore, the various attachment locations had different amplitudes and directions of the marker displacements (Figs. 4 and 5). The cranial thigh markers (CDT, CMT, and CPT) exhibited the greatest STA variation in terms of the *rmsd* and Δ*p*_*max*_ values (Fig. 4 and Table 1). The primary STA component appeared in the proximal/distal (P/D) direction and accounted for approximately 70% of the total magnitude. The cranial thigh markers were typically displaced distally when the stifle joint was flexed from the extended position (Fig. 5). The lateral thigh markers exhibited a smaller STA (Table 1), and the cranial/caudal (C/C) component was generally the primary error component, except at the LDT marker (Fig. 5). The LFC marker exhibited an opposite displacement to that of the other four lateral markers. Lateral crus markers closer to the stifle joint (FH, LPC, and LMC) exhibited a greater STA (Fig. 4), while the remaining markers appeared to move locally with an *rmsd* of < 3.5 mm (Table 1). The LPC and LMC markers moved cranially, and the FH marker moved distally and cranially when the stifle was flexed from the extended position (Fig. 5).

**Table 1:** Soft tissue artifacts of the skin markers

Segment	Marker	*rmsd* (mm)	*rmsd*_*x*_ (mm)	*rmsd*_*y*_ (mm)	*rmsd*_*z*_ (mm)	*∆p*_*max*_ (mm)	*∆p*_*x*_ (mm)	*∆p*_*y*_ (mm)	*∆p*_*z*_ (mm)
Thigh	GT	6.1 ± 1.3	5.5 ± 1.4	1.7 ± 0.7	1.7 ± 0.5	19.2 ± 4.2	18.0 ± 4.4	5.4 ± 2.2	6.7 ± 1.9
	LPT	4.4 ± 1.2	3.6 ± 1.5	1.2 ± 0.5	1.8 ± 0.5	14.1 ± 4.0	12.8 ± 4.5	4.0 ± 1.5	6.8 ± 2.0
	LMT	3.6 ± 1.4	3.0 ± 1.6	1.2 ± 0.5	1.4 ± 0.5	13.2 ± 5.1	11.7 ± 5.3	4.1 ± 1.5	5.7 ± 2.1
	LDT	3.8 ± 1.1	2.3 ± 1.0	1.4 ± 0.9	2.4 ± 0.9	13.1 ± 4.0	9.0 ± 4.2	4.8 ± 3.0	8.7 ± 3.0
	LFC	5.5 ± 1.3	3.9 ± 1.8	1.2 ± 0.7	3.1 ± 1.4	16.6 ± 3.7	12.2 ± 5.0	4.0 ± 2.0	10.2 ± 4.2
	CDT	8.7 ± 1.5	3.5 ± 0.8	7.3 ± 1.5	2.6 ± 1.3	25.7 ± 4.4	10.9 ± 2.7	22.0 ± 4.3	8.9 ± 4.0
	CMT	8.6 ± 1.5	2.5 ± 0.7	7.4 ± 1.5	3.1 ± 1.3	25.4 ± 3.8	8.2 ± 2.2	22.1 ± 4.2	10.6 ± 3.7
	CPT	9.1 ± 1.6	1.4 ± 0.7	7.7 ± 1.5	4.4 ± 1.2	27.4 ± 3.8	5.3 ± 2.4	23.2 ± 4.0	14.9 ± 3.3
Crus	FH	5.7 ± 2.6	3.7 ± 2.6	3.3 ± 1.6	2.2 ± 1.3	16.7 ± 7.7	12.1 ± 7.9	9.4 ± 4.3	7.2 ± 3.7
	LPC	9.8 ± 2.4	9.2 ± 2.2	1.9 ± 1.4	2.6 ± 0.9	28.7 ± 7.2	27.0 ± 6.8	5.8 ± 3.6	8.8 ± 2.9
	LMC	8.5 ± 1.7	7.9 ± 1.7	1.7 ± 1.1	2.0 ± 0.7	24.4 ± 4.5	23.3 ± 4.7	5.3 ± 2.8	6.2 ± 1.4
	LDC	3.2 ± 0.7	2.7 ± 0.6	1.1 ± 0.6	1.1 ± 0.3	9.7 ± 2.0	8.8 ± 1.9	3.6 ± 1.5	3.9 ± 0.7
	LM	1.8 ± 0.4	1.2 ± 0.5	0.9 ± 0.3	0.9 ± 0.1	5.8 ± 1.4	4.6 ± 1.7	3.3 ± 0.9	3.6 ± 0.4
	CDC	1.8 ± 0.4	1.1 ± 0.6	1.0 ± 0.4	0.8 ± 0.3	5.8 ± 1.3	4.1 ± 1.8	3.6 ± 1.2	3.3 ± 1.0
	CMC	2.2 ± 0.7	1.1 ± 0.6	1.0 ± 0.4	1.5 ± 0.7	7.2 ± 2.2	4.1 ± 2.0	3.8 ± 1.2	5.3 ± 2.0
	DTC	2.9 ± 1.0	1.3 ± 0.7	1.3 ± 0.7	2.0 ± 0.8	8.8 ± 2.8	4.5 ± 1.6	4.8 ± 2.6	6.7 ± 2.2
	PTC	3.5 ± 1.2	1.5 ± 0.9	2.2 ± 1.0	2.0 ± 0.8	10.9 ± 4.3	4.9 ± 2.8	7.9 ± 3.9	6.9 ± 2.1

The soft tissue artifacts of the markers are shown as the total magnitude of the root-mean-square amplitude (*rmsd*) and the *rmsd* along each anatomical axis (*rmsd*_*c*_, where *c* represents the *x-*, *y-,* and *z-*axes). The corresponding values for the total magnitude of the peak-to-peak amplitude (∆*p*_*max*_) and along the three anatomical axes (∆*p*_*c*_) were calculated. Data are shown as the mean ± standard deviation. Marker locations are shown in Fig. 1

**Fig. 6:**
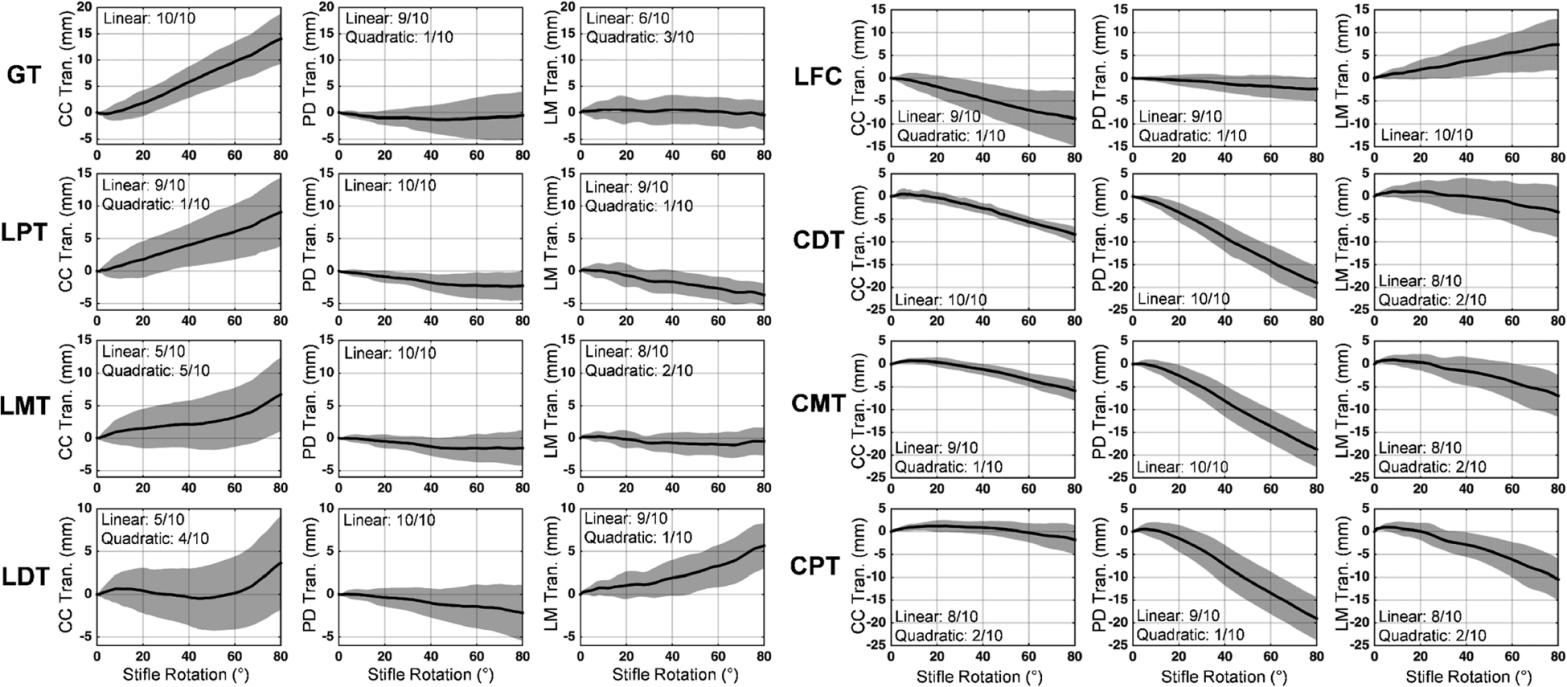
Patterns of the soft tissue artifacts of the thigh markers relative to the stifle rotation. The mean (black) and standard deviation (gray) values for the marker displacements along the cranial/caudal (CC), proximal/distal (PD), and lateral/medial (LM) axes of the femoral bone coordinate system, relative to the stifle rotation. The stifle rotation is defined as the angle of the stifle flexion with respect to the most extended position (i.e., the ending joint angle)

**Fig. 7:**
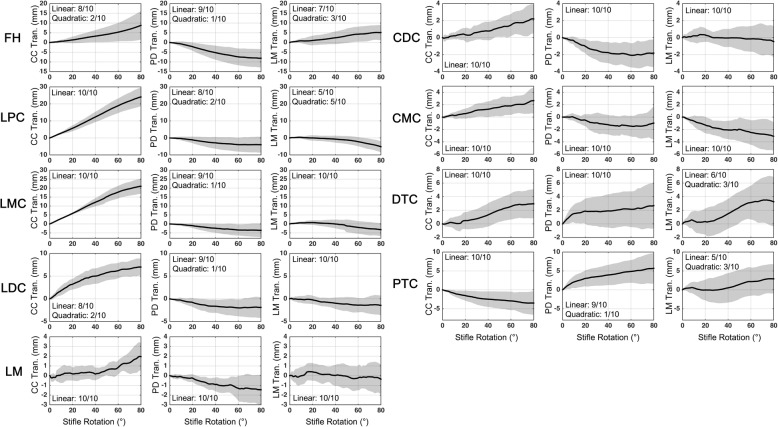
Patterns of the soft tissue artifacts of the crus markers relative to the stifle rotation. The mean (black) and standard deviation (gray) values for the marker displacements along the cranial/caudal (CC), proximal/distal (PD), and lateral/medial (LM) axes of the tibial bone coordinate system, relative to the stifle rotation. The stifle rotation is defined as the angle of the stifle flexion with respect to the most extended position (i.e., the ending joint angle)

The marker STA patterns revealed that most markers were primarily classified into linear and quadratic patterns for the three anatomical directions (Figs. 6 and 7, Additional file 1). For each component of each marker, at least one-half of the subjects exhibited a linear STA against the stifle rotation angles. For the thigh markers, the major error component for each marker (e.g., the P/D direction for CMT) was consistently linear with respect to the stifle rotation (in > 90% of the subjects) (Fig. 6), with the exceptions of the LMT and LDT markers. The C/C component of these two markers was closer to the quadratic STA pattern (Fig. 6), in which only one-half of the subjects exhibited linear STA patterns. The major STA components for the crus markers were also linear with respect to stifle rotation (Fig. 7). While the patterns of the lateral/medial (L/M) STA components for some markers appeared to be closer to quadratic or higher-order polynomial curves, the STA in the L/M direction were not major sources of error, as the ranges were generally < 5 mm or much less than that of the primary STA component.

Significant effects of the STA on the calculated stifle joint angles were observed (Table 2). During 0–50% of the motion task (i.e., from the full flexion position to a stifle flexion angle of approximately 90°), the SM-determined stifle flexion angles were significantly smaller than the VM-determined values. During the same task interval, the SM-determined internal rotations were significantly greater than the VM-determined values. During 0–30% of the motion task, the SM-determined adduction angles were significantly greater than the VM-determined values (Table 2).

**Table 2:** Effects of soft tissue artifacts on the estimation of joint angles

	Flexion/extension (°)				Adduction/abduction (°)				Internal/external rotation (°)			
Cycle	SM	VM	(SM–VM)	*p*-value	SM	VM	(SM–VM)	*p*-value	SM	VM	(SM–VM)	*p*-value
0%	141.1 ± 7.4	147.6 ± 8.5	−6.6 ± 2.6	< 0.001*	4.3 ± 7.3	−0.9 ± 5.7	5.1 ± 4.1	0.004*	35.5 ± 12.0	9.2 ± 13.1	26.4 ± 12.0	< 0.001*
10%	132.1 ± 8.9	137.9 ± 9.7	−5.8 ± 2.5	< 0.001*	5.1 ± 8.1	−0.1 ± 6.7	5.2 ± 4.5	0.005*	31.7 ± 11.6	9.4 ± 12.2	22.3 ± 11.1	< 0.001*
20%	121.6 ± 10.4	126.8 ± 11.2	−5.2 ± 2.3	< 0.001*	6.0 ± 8.8	0.8 ± 8.1	5.1 ± 4.8	0.009*	27.0 ± 11.1	9.4 ± 10.7	17.6 ± 10.4	0.001*
30%	110.1 ± 12.0	114.5 ± 12.8	−4.4 ± 2.2	< 0.001*	6.7 ± 9.3	2.5 ± 9.2	4.2 ± 5.6	0.043*	21.7 ± 10.8	8.3 ± 9.3	13.4 ± 9.6	0.002*
40%	98.6 ± 12.8	102.1 ± 13.6	−3.5 ± 2.3	0.001*	6.8 ± 9.6	3.8 ± 9.8	3.0 ± 6.6	0.184	16.3 ± 10.3	7.0 ± 7.7	9.3 ± 8.3	0.006*
50%	88.1 ± 12.9	90.4 ± 13.6	−2.4 ± 2.2	0.008*	6.8 ± 9.3	4.8 ± 10.0	1.9 ± 6.6	0.384	11.9 ± 9.6	6.0 ± 6.3	6.0 ± 6.3	0.015*
60%	78.7 ± 12.7	79.9 ± 13.4	−1.2 ± 2.2	0.128	6.4 ± 9.1	5.5 ± 9.6	0.9 ± 6.4	0.672	8.4 ± 9.1	5.2 ± 5.9	3.2 ± 4.7	0.058
70%	70.5 ± 12.2	70.7 ± 13.0	−0.3 ± 2.4	0.741	6.1 ± 8.5	5.8 ± 9.3	0.3 ± 5.9	0.894	5.8 ± 9.1	4.8 ± 6.5	1.0 ± 3.5	0.392
80%	63.9 ± 11.7	63.6 ± 12.7	0.3 ± 2.7	0.727	5.8 ± 8.1	6.0 ± 8.7	−0.2 ± 4.8	0.903	3.8 ± 9.5	4.9 ± 7.5	−1.1 ± 3.3	0.323
90%	59.0 ± 10.6	58.5 ± 12.2	0.5 ± 2.9	0.623	6.0 ± 7.6	6.3 ± 8.5	−0.3 ± 4.4	0.856	2.3 ± 10.1	4.4 ± 8.2	−2.1 ± 3.4	0.078
100%	55.9 ± 10.6	55.7 ± 12.5	0.2 ± 2.9	0.841	6.4 ± 7.3	6.8 ± 8.4	−0.4 ± 4.3	0.757	0.9 ± 10.4	3.5 ± 8.3	−2.6 ± 3.6	0.051

The stifle flexion/extension, adduction/abduction, and internal/external rotation values based on the skin markers (SM) and virtual markers (VM) were computed. Data are shown as the mean ± standard deviation across subjects. The data points were normalized for a complete motion cycle and are expressed as percentages. Differences between the two measurement outcomes were also calculated by subtracting the VM results from the SM results. Asterisks indicate significant differences (*p* < 0.05)

## Discussion

The present study assessed the STA of the canine hindlimb by combining radiological images and marker trajectories from a motion capture system. Due to technical limitations, previous studies could only use inter-marker distance changes to indicate STA during a gait cycle [18, 19]. These distance changes did not provide information on how the individual markers move relative to the underlying bone, nor did they quantify the effects of STA on angles. With the current approach, the present study bridges this gap by directly quantifying individual marker STA, revealing that the opposite direction of the cranial/caudal displacement between the GT and LFC markers (Fig. 5) could explain why the femoral length changes during gait.

During stifle extension, the craniolateral thigh and crus markers generally exhibited different STA for different amplitudes and directions. The thigh marker STA were greater than the crus marker STA, with the exception of the FH, LPC, and LMC markers (Table 1). In addition, the cranial thigh markers exhibited the greatest errors in the P/D direction, which is in agreement with the STA distribution in the human thigh during open kinetic chain flexion of the knee [10]. The greater STA in the cranial thigh may be associated with the abundant soft tissues overlying the femur (e.g., the quadriceps muscle and subcutaneous tissues), which would indicate that the markers moved with the skin surface as a result of tissue stretching and sliding during stifle rotation. The lateral markers exhibited quite different STA directions and generally had smaller error quantities than the cranial markers. Although the current motion task was restricted to stifle extension, it was difficult to completely avoid a slight swing of the thigh during data acquisition. For example, the fluoroscopic images indicate that slight femoral elevations were detected when the stifle was bent to high flexion. This could have led to an increase of hip flexion and an overestimation of the stifle motion-related STA near the hip (e.g., at the GT marker) [17].

The crus FH, LPC, and LMC markers were remarkably displaced along the C/C direction (Fig. 5), which possibly resulted from the squeezing of the profound muscle tissues in the caudal aspects of the thigh and crus during flexion. This could have produced a forward movement of lateral skin surface. However, the remaining crus markers moved locally with an *rmsd* of < 3.5 mm. In this context, the tibialis cranialis muscle is responsible for tarsal flexion and would be expected to contribute to the cranial crus marker STA. However, the present study restricted hock joint rotation using a custom-made jig, which minimized the effects of this muscle on the STA. Therefore, studies of STA during functional activities (e.g., gait) that involve multiple joint motions should consider the motion of the hip and hock and their effects on markers placed near the related muscles.

The present study involved only passive stifle extension, which did not replicate dynamic conditions. While this is a limitation, passive stifle motion provided an ideal opportunity to examine the relationships between stifle rotation and the STA amplitudes as it minimized the influence of the adjacent joints and STA variation components associated with soft tissue wobbling and muscles activities. A linear regression analysis was used to assess the linearity of the marker STA, which identified the lowest-degree polynomial function that accounted for the general curve of the STA patterns. This approach revealed largely linear relationships between the stifle rotations and the STA amplitudes, and this may facilitate the application of a generalized linear STA model architecture based on adjacent joint motions [16] to canine motion analysis.

The marker STA in the craniolateral segment could result in significant errors in stifle joint angle estimation. Based on the cluster templates (8 thigh markers and 9 crus markers), the stifle flexion angle was underestimated (Table 2) as a result of the cranially tilted thigh. Thus, the distally displaced cranial markers, the cranially displaced lateral markers, and the caudally displaced LFC marker contributed to thigh misalignment during stifle flexion (Fig. 5). At the same poses, misalignment of the crus with respect to the true pose, which occurred in the coronal plane, caused by the slightly distal displacement of the lateral markers (Fig. 5), probably led to an overestimated adduction angle for the stifle joint. The STA had the greatest effects on the internal rotation estimation (Table 2), and the misalignment of the thigh and crus both contributed to the overestimated internal rotation. During high flexion of the stifle, the medial tilt and internal rotation of the thigh would result in a more externally oriented floating axis in the joint coordinate system [28]. Thus, the medially rotated crus and the externally rotated joint axis would generate overestimated internal rotations of the stifle joint.

The current estimations revealed that stifle kinematics were affected by the STA, with significant differences in the calculated joint angles mainly occurring when the flexion angles were greater than 90°, a value beyond those found during typical walking and stair ascending [21]. These effects suggest that “stifle flexion-induced STA” do not erroneously affect the measurements of typical ambulation, except for activities involving high stifle flexions (e.g., sitting) [21]. While the reported data revealed hidden relationships between STA and passive stifle rotations and helped to clarify their effects on the calculated stifle angles, it is probably not appropriate to apply the current findings to previous canine gait studies directly. For dynamic activities, STA could be induced by muscle contractions, mass wobbling and tendon displacements. To faithfully describe STA patterns and better reflect STA effects on the measurements of stifle kinematics during voluntary ambulation involving multiple joint motions and inertial effects, an additional study conducted in accordance with canine gait analysis conventions is needed. In the current study, a large number of markers, including those used in regular gait analysis, were used to quantify the 3-D STA patterns at various locations. For future use in basic research and clinical applications, only a subset of the markers is needed. Systematic studies on the effects of marker cluster combinations of 3–5 markers per segment [22] on the calculated joint kinematics would be needed.

One of the limitations of the current study is that mixed-breed dogs of various ages were used, although the dogs were of similar body build. Since breed and age variations may affect the state of hydration and consequently of skin mobility, further study will be needed to quantify their effects on the STA and stifle joint kinematics [29]. A second limitation is the presence of factors that may introduce subtle changes in the STA pattern. The skin region in contact with the medial, cranial, and caudal sides of hammock openings was likely affected, resulting in reduced STA amplitudes of the analyzed markers (e.g., CPT) and the underestimation of STA-induced errors. Anesthesia-induced muscle relaxation may also introduce subtle changes in passive stifle kinematics and the consequent marker STA patterns.

## Conclusions

This study provides the first quantitative data regarding 3-D STA in the craniolateral aspects of the canine thigh and crus during passive stifle motion. The data obtained revealed considerable marker displacements relative to the underlying bones, especially in the cranial aspect of the thigh and the proximal-lateral region of the crus; these displacements were generally linear against the rotation of the stifle joint. These thigh and crus STA are expected to result in underestimated flexion angles and overestimated adduction and internal rotation angles when the stifle is flexed to > 90°, suggesting that “stifle flexion-induced STA” do not erroneously affect the measurements of the joint kinematics of typical ambulation patterns, such as gait. On the other hand, these findings can provide a reference for marker selection in canine motion analysis for similar motion tasks and can aid in clarifying the relationship between STA patterns and stifle kinematics, which may contribute to the development of STA models and compensation techniques for canine motion analysis.

## Additional file

Additional file 1: Linear regression equations for the soft tissue artifacts of the markers. Linear regression equations were computed for marker displacement components greater than 10 mm with the stifle rotation angle as regressor. The stifle rotation is defined as the angle of the stifle flexion with respect to the ending joint angle. (DOCX 34 kb)

## Abbreviations

2-D: Two-dimensional
3-D: Three-dimensional
Add/Abd: Adduction/Abduction
C/C: Cranial/Caudal
CDC: Cranial-distal crus marker
CDT: Cranial-distal thigh marker
CMC: Cranial-middle crus marker
CMT: Cranial-middle thigh marker
CPT: Cranial-proximal thigh marker
CT: Computed tomography
DTC: Distal tibial crest marker
FH: Fibular head marker
Flex/Ext: Flexion/Extension
FOV: Field of view
GT: Greater trochanter
IR/ER: Internal/External rotation
L/M: Lateral/Medial
LDC: Lateral-distal crus marker
LDT: Lateral-distal thigh marker
LFC: Lateral femoral epicondyle marker
LM: Lateral malleolus marker
LMC: Lateral-middle crus marker
LMT: Lateral-middle thigh marker
LPC: Lateral-proximal crus marker
LPT: Lateral-proximal thigh marker
MFC: Medial femoral epicondyle marker
MM: Medial malleolus marker
P/D: Proximal/Distal
PTC: Proximal tibial crest marker
SD: Standard deviation
SM: Skin marker
STA: Soft tissue artifacts
VM: Virtual marker
