# Supplementary material for: Quantification of three-dimensional soft tissue artifacts in the canine hindlimb during passive stifle motion
Source: BMC Vet Res. 2018 Dec 7;14:389. doi: 10.1186/s12917-018-1714-7 (PMC6284316; doi:10.1186/s12917-018-1714-7)
Supplement: Supplementary file 1 — Linear regression equations for the soft tissue artifacts of the markers. Linear regression equations were computed for marker displacement components greater than 10 mm with the stifle rotation angle as regressor. The stifle rotation is defined as the angle of the stifle flexion with respect to the ending joint angle. (DOCX 34 kb) [file 12917_2018_1714_MOESM1_ESM.docx]

| Marker | Cranial-Caudal | Proximal-Distal | Lateral-Medial |
| --- | --- | --- | --- |
| GT | $E(r)=0.18r-1.36$ | - | - |
| LPT | $E(r)=0.10r-0.10$ | - | - |
| LMT | $E(r)=0.07r-0.06$ | - | - |
| LFC | $E(r)=-0.14r-0.91$ | - | $E(r)=0.07r+0.58$ |
| CDT | $E(r)=-0.12r+1.61$ | $E(r)=-0.26r+1.21$ |  |
| CMT | - | $E(r)=-0.26r+2.08$ | $E(r)=-0.09r+1.85$ |
| CPT | - | $E(r)=-0.27r+3.02$ | $E(r)=-0.13r+2.08$ |
| FH | $E(r)=0.12r-0.97$ | - | - |
| LPC | $E(r)=0.31r-0.22$ | - | - |
| LMC | $E(r)=0.25r+1.36$ | - | - |
